# StackZDPD: a novel encoding scheme for mass spectrometry data optimized for speed and compression ratio

**DOI:** 10.1038/s41598-022-09432-1

**Published:** 2022-03-30

**Authors:** Jinyin Wang, Miaoshan Lu, Ruimin Wang, Shaowei An, Cong Xie, Changbin Yu

**Affiliations:** 1 Zhejiang University, Hangzhou, 310058 China; 2 School of Life Science, Westlake University, Hangzhou, 310023 China; 3 School of Engineering, Westlake University, Hangzhou, 310023 China; 4 Fudan University, Shanghai, 200438 China; 5 College of Artificial Intelligence and Big Data for Medical Science, Shandong First Medical University, Jinan, 250117 China; 6 Carbon Silicon (Hangzhou) Biotechnology Co., Ltd, Hangzhou, 310030 China

**Keywords:** Metabolomics, Proteomics

## Abstract

As the pervasive, standardized format for interchange and deposition of raw mass spectrometry (MS) proteomics and metabolomics data, text-based mzML is inefficiently utilized on various analysis platforms due to its sheer volume of samples and limited read/write speed. Most research on compression algorithms rarely provides flexible random file reading scheme. Database-developed solution guarantees the efficiency of random file reading, but nevertheless the efforts in compression and third-party software support are insufficient. Under the premise of ensuring the efficiency of decompression, we propose an encoding scheme “Stack-ZDPD” that is optimized for storage of raw MS data, designed for the format “Aird”, a computation-oriented format with fast accessing and decoding time, where the core compression algorithm is “ZDPD”. Stack-ZDPD reduces the volume of data stored in mzML format by around 80% or more, depending on the data acquisition pattern, and the compression ratio is approximately 30% compared to ZDPD for data generated using Time of Flight technology. Our approach is available on AirdPro, for file conversion and the Java-API Aird-SDK, for data parsing.

## Introduction

Currently, the most widely used format for long-term storage in mass spectrometry (MS) is original proprietary vendor format. Pitifully, this kind of format cannot meet the needs of cross-platform computing and is lack of adaptability to kinds of software. The Proteomics Standards Initiative (PSI) established a standardized Extensible Markup Language (XML) representation for raw data interchange in mass spectrometry (MS) in 2008^1^, called “mzML”, further building upon concepts defined in mzData and mzXML^2^. mzML is now the pervasive format for interchange and deposition of raw MS proteomics and metabolomics data^3^. However, in the design of “mzML”, like all text-based XML file formats, numeric spectrum data should be converted into text strings using Base64 encoding^4^. Although the numeric data can be Zlib^5^ compressed before encoding, the sizes of mzML files are still 4 to 18-fold higher than vendor formats. That is, the memory of a typical desktop computer cannot handle the analysis of even a single proteomics file. Research projects on MS data compression have sprung up, to address difficulties arise in usage of mzML format for large-volume data analysis.

One approach for biological sampled, high-throughput data analysis is random access file reading, allowing specific data to be efficiently extracted, thus there is no need to read an entire file into memory. HDF5^6^ (Hierarchical Data Format version 5) is a binary format developed by the National Center for Supercomputing Applications (NCSA) for the storage and organization of large amount of data. Mz5^5^ and Toffee^7^ are both developed on HDF5 technology. HDF5 allows multidimensional arrays of data elements of a specific type (e.g., integer, floating point, characters, strings, or a collection of these organized as compound types), bringing an average file size reduction of 54% and increases linear read and write speeds three–fourfold according to the comparison of mz5 and mzML. Ranjeet proposed an HDF5 file format “mzMLb”^8^ that is optimized for both read/write speed and storage of the raw mass spectrometry data. Another similar database-based solution is mzDB^9^, which is developed upon the lightweight SQLite relational database technology. In comparison with XML formats, mzDB saves 25% of storage space. In addition, data access times are cut in half or less when compared to mzML, counting on the data access mode. However, one of the bottlenecks of these database-based format is that data written in mz5 or mzDB precludes a Java implementation using the corresponding Java application programming interface (API) as compound structures are extremely slow to access with API.

Different from mz5, mzDB, and Toffee, Numpress^10^ and MassComp^11^ are encoding schemes for mzML, focusing on a novel algorithm to compress the binary data in the mzML file before Base64 encoding. Numpress was described to compress mzML file size by around 61%, even approximately 86% if additionally Zlib compressed. Though Numpress have better performance in compression than database-based formats, there is no flexible random file reading scheme provided in the design of Numpress. Not alone, so is MassComp.

Aird^12^ is developed considering both efficient data reading and compression performance. It is a computation-oriented format targets for fast accessing and decoding time, providing controllable precision and multiple index strategies to reconstruct the mass spectrum file. For metadata, Aird uses JavaScript Object Notation (JSON) rather than XML to reach a lightweight metadata file with similar readability and extensibility as XML. Also, JSON has better performance in web-side development. That is JSON can better adapt to the cloud service support of MS data. A JSON Aird Schema (https://github.com/CSi-Studio/Aird-SDK/blob/1.1/AirdMetaData.json) is provided and detailed description for every field in the Aird metadata is public on the GitHub page. For spectrometry data, Aird applies a novel compression method ZDPD^12^ (Zlib, diff, pFor and delta). Floating-point mass-to charge ratio (m/z) array is converted to ordered integer array to use FastPfor^13^ to compress. FastPfor is a library with integer compression schemes. It is broadly applicable to the compression of arrays of 32-bit integers. The library exploits the Single Instruction Multiple Data (SIMD)^13^ instructions to achieve faster compression and decompression. After additionally Zlib compressing, Aird can reduce 53% of its volume when using 1 part per million (ppm) as the precision requirement, but only takes 33% of the time for decoding, compared to using Zlib only.

On the basis of ZDPD, we developed Stack-ZDPD (SZDPD) to achieve higher compression ratio. Considering that pFor compresses smaller and more repetitive integer arrays better, m/z arrays of several spectrums are joined before compression. Storage volume of m/z array reduces at the cost of the addition of tag array, where each m/z corresponds to a tag of a spectrum for decoding. Taken together, when appropriate layers of spectrums are stacked, the overall compression rate can be improved.

## Results

### Best practice of layer number choice

**Figure 1:**
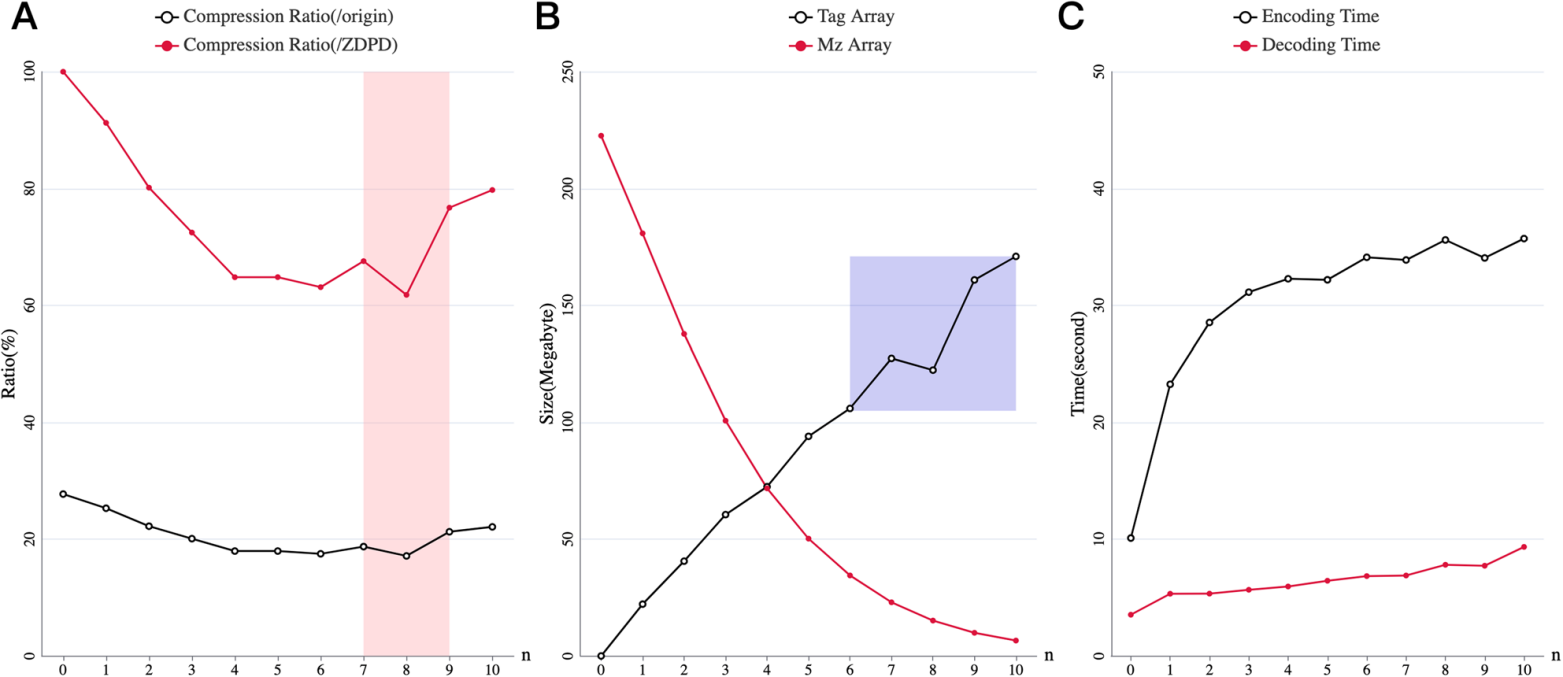
(**A**) Curve of MS data compression ratio-change along with n. The black line with empty circle is generated from size of data compressed with SZDPD divided by size of original arrays, and for red curve with circle, divided by size of ZDPD-compressed arrays. (**B**) The size of tag array and m/z array varies with n, respectively. Data compressed with SZDPD is composed of tag array and m/z array, and the change curves of the two arrays are represented by black line and red line respectively. (**C**) The growth curve of encoding and decoding time as n increases.

We firstly analyze the effect of the number of spectra in a stack on the compression rate of m/z array applied SZDPD. Given that tag of 2^n^ spectrums require n bits for storage, MS data of the first 5 MS2 blocks in File 8 (shown in Table 1) is used to test SZDPD of different n, and the comparison result is shown in Fig. 1. Overall, as n increases, SZDPD performs better first and then worse. As for its improvement relative to ZDPD, the pattern is the same. The area marked with red shadow in Fig. 1. A should be paid extra attention for the sum of the size of m/z and tag array decreases when n goes from 7 to 8. The compression ratio comes to the minimum when n equals to 8, mainly benefits from the reduction of tag array size, which is marked with the blue rectangle in Fig. 1B. Merging m/z arrays results in smaller deltas, and in turn leads to a higher compression ratio for pFor, which can be seen from the decreasing size of m/z array as number of spectra in a stack grows. When n is equal to 0, a stack contains one spectrum, which we can regard as compressing with ZDPD. In Fig. 1C, decoding time is little changed as n grows. In analyzing software widely used in field of MS like OpenSWATH^14^, MZMine^15^, XCMS^16^ and so on, extracted ion chromatogram (XIC) is heavily used during calculation. Though encoding time is markedly increased, we tend to focus more on decompression time, for the decoding frequency is much higher than encoding.

**Table 1:** The raw MS data files used in validation, together with MS instrument and method information.

File No	MS data file name	Type	Vendor	Instrument
1	0530_BG_293T_1_SWATH_1	SWATH DIA	SCIEX	Triple TOF5600
2	20180722_L929_test_DDA_1	DDA	SCIEX	Triple TOF5600
3	QE-HFX-20190719_50cm_60min_OFe4_2	SWATH DIA	Thermo	Orbitrap exactive
4	QE-HFX-20190719_50cm_60min_Fr1	DDA	Thermo	Orbitrap exactive
5	S8184TPST_01	SWATH DIA	Thermo	Orbitrap exactive
6	Negative_000333	SWATH DIA	Thermo	Orbitrap exactive
7	SampleA_1	SWATH DIA	SCIEX	Triple TOF6600
8	HYE110_TTOF6600_32fix_lgillet_I160308_001	SWATH DIA	SCIEX	Triple TOF6600
9	HYE110_TTOF6600_64fix_lgillet_I160310_001	SWATH DIA	SCIEX	Triple TOF6600
10	HYE124_TTOF5600_32fix_lgillet_L150206_001	SWATH DIA	SCIEX	Triple TOF5600
11	HYE124_TTOF5600_64var_lgillet_L150206_007	SWATH DIA	SCIEX	Triple TOF5600
12	Set 1_F2	DDA	Agilent	6550 iFunnel Q-TOF
13	SA1	DDA	Thermo	QE HF
14	LFQ_Orbitrap_AIF_Human_01	AIF DIA	Thermo	Orbitrap QE HF-X
15	LFQ_Orbitrap_DDA_Human_01	DDA	Thermo	Orbitrap QE HF-X
16	LFQ_TTOF5600_DDA_Human_01	DDA	SCIEX	Triple TOF5600
17	LFQ_TTOF5600_SWATH_Human_01	SWATH DIA	SCIEX	Triple TOF5600
18	LFQ_TTOF6600_DDA_Human_01	DDA	SCIEX	Triple TOF6600
19	LFQ_TTOF6600_SWATH_Human_01	SWATH DIA	SCIEX	Triple TOF6600

Files 1–2 are from ref^17^, data files 3–4 are from ref^18^, 5 is from ref^19^, 6 is from ref^20^, 7 and 13 is from ref^21^, 8–11 are from ref^22^, 12 is from ref^23^, and finally 14–19 are from ref^24^.

### Comparison with several compression algorithms

**Figure 2:**
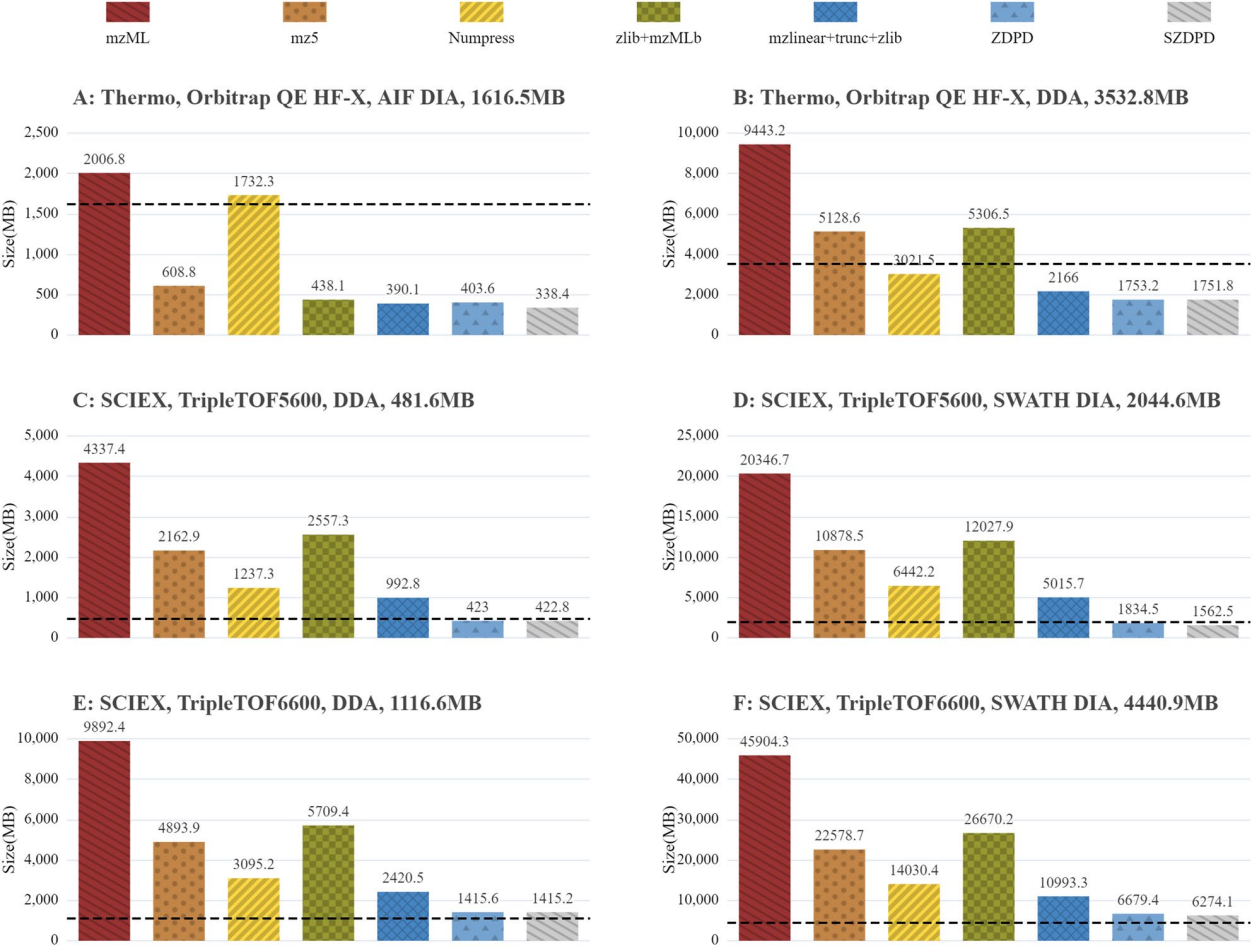
Summary data showing file sizes for all data sets using the seven formats with different compression combinations spanning both lossless and lossy configurations. File 14–19 are used and mz is stored of 5 decimal points in Aird format. The original vendor file sizes are represented by the horizontal dashed line.

Relative to vendor format, we compared the compression performance of mzML, mz5, Numpress, mzMLb, ZDPD and SZDPD, and the result is shown in Fig. 2. To make the comparison more accurate, we selected mass spectrometry files of the same sample produced by different instruments. Whether data dependent acquisition (DDA) file or data independent acquisition (DIA) file, file sizes are significantly smaller when stored in the Aird format compared to mzML, mz5, Numpress and mzMLb, even smaller than the vendor’s format.

**Figure 3:**
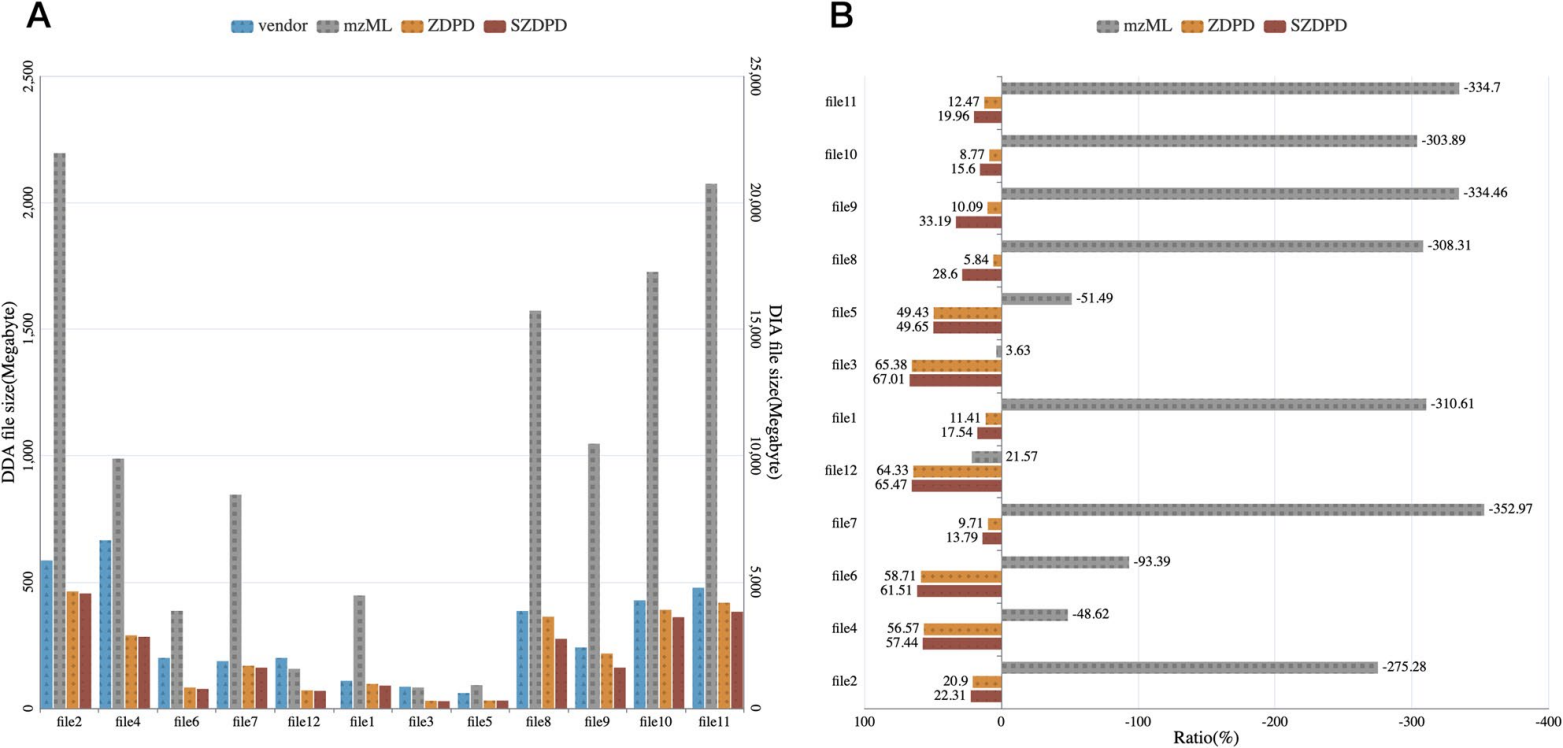
(**A**) File size of data stored with different format. For file 2, 4, 6, 7 and 12, the data acquisition mode is DDA, and the rest files are of SWATH/DIA mode. (**B**) Compression ratio of mzML, ZDPD and SZDPD. The ratio is calculated by [size(vendor) − size(x)]/size(vendor)*100%, where x is mzML, ZDPD and SZDPD respectively.

To test different databases, we did a comparison experiment with another 12 files and the result is shown in Fig. 3.

Files applying SZDPD are further compressed on the basis of ZDPD, and the promotion is better in DIA files than DDA. In order to see the compression effect more clearly, we calculated the compression ratio of the new format compared to the vendor files (shown in Fig. 3B). Overall, compared with ZDPD, the compression performance of SZDPD is improved. In several files (file 7, 8, 9), the promotion effect is more obvious. Given that the test files are from different databases, the main difference in the compression of different files is the instrument that generated the data. We focus on how SZDPD improves ZDPD, and the results show that SZDPD is more suitable for SCIEX files than other vendors. Further, among SCIEX vendor files, the compression promotion of data produced by TOF6600 is better than TOF5600, and the compression rate can be increased to about 30% provided that ZDPD has a good record of compressing vendor files by about 9%.

### Tradeoff between information loss and compression ratio

**Figure 4:**
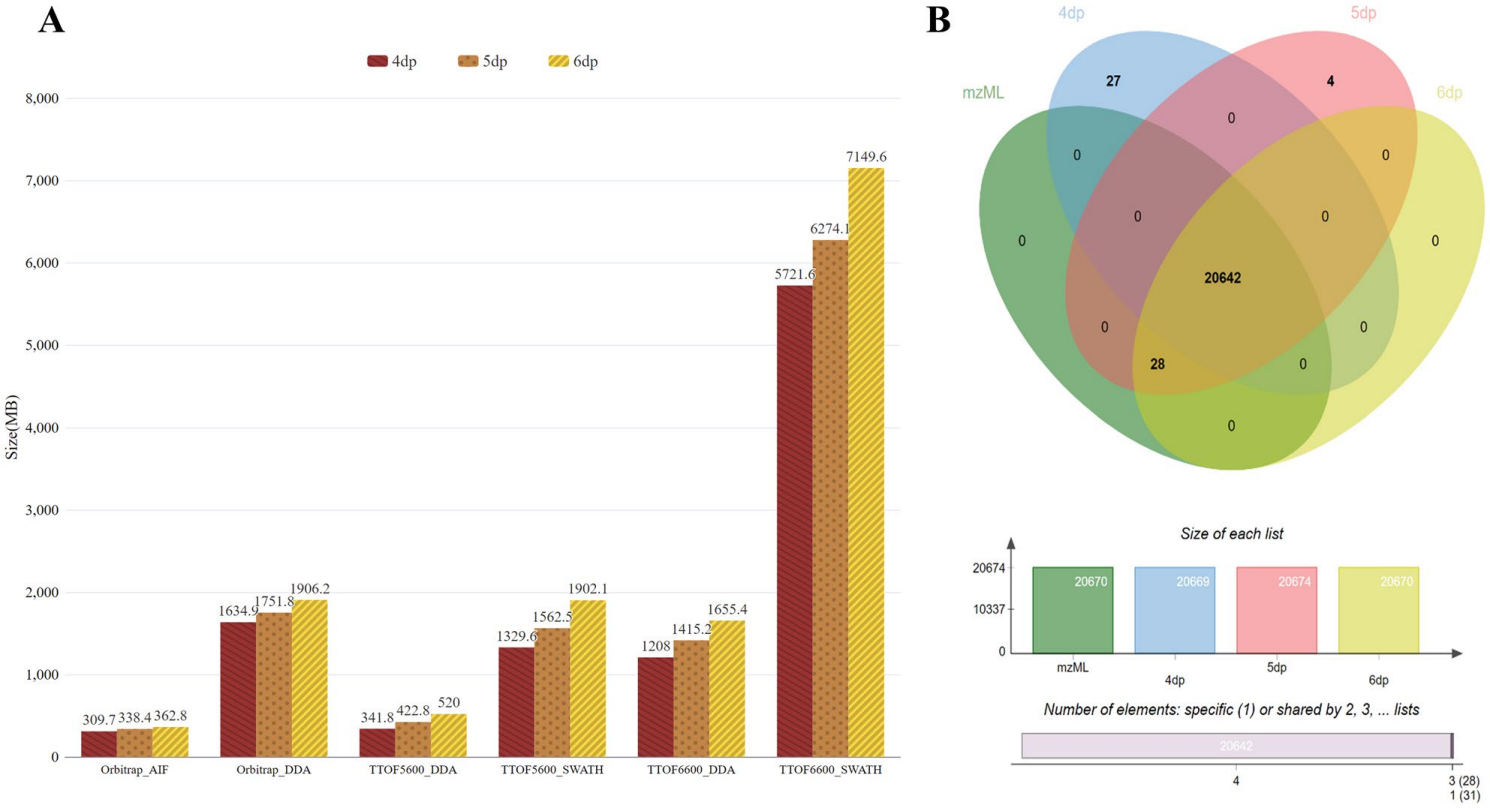
(**A**) Files with mz stored at 4, 5, and 6 decimal points size comparison. (**B**) Peak number comparison of mzML file and Aird (4, 5, 6 decimal points stored) files, after ADAP chromatogram building in MZmine3. Vendor file 13 is used.

We have supported the MZmine^15^ software with mass spectrometry files in Aird format. We use the raw data import tool in MZmine3 and compare the file import time of mzML, Numpress and Aird. As a result, MZmine3 imports the Aird (SZDPD_dp5) format one-third of the time it takes to import the mzML format and one-half the time to the Numpress. To analyze the tradeoff between information loss and compression ratio. we have done experiments on accuracy testing. The SZDPD algorithm is applied to save the mass spectrum file, where the mz is stored at 4, 5, and 6 decimal points respectively, and the file size and XIC results are compared, shown in Fig. 4. The results after ADAP^25^ chromatogram building (Fig. 4B) show that Aird files stored in 6 dp precision are exactly the same as mzML, 5 dp precision storage is almost the same, and 4 dp precision storage is slightly different.

**Figure 5:**
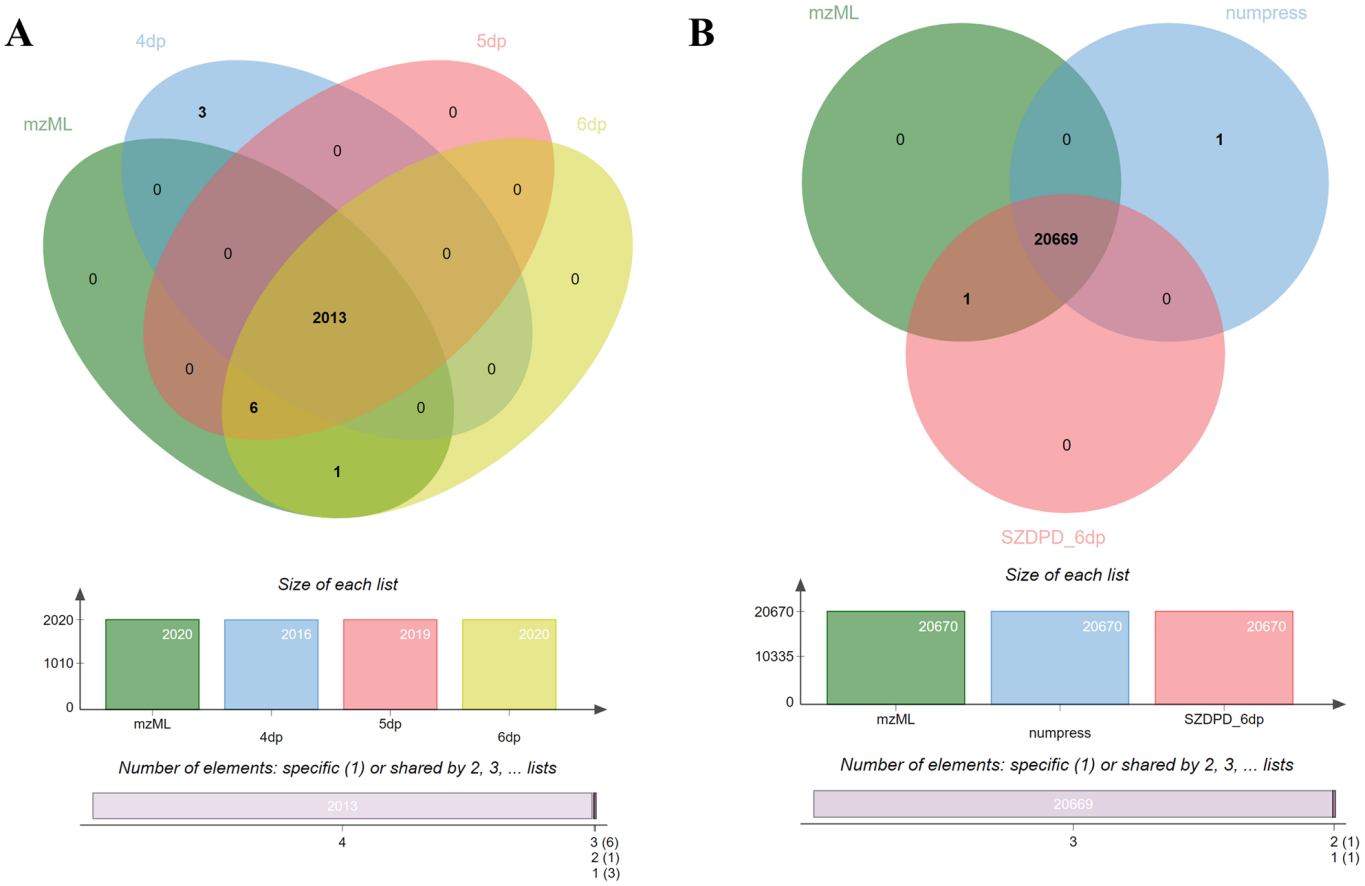
(**A**) Peak number comparison of mzML file and Aird (4, 5, 6 decimal points stored) files, after ADAP chromatogram building in MZmine3. Vendor file 7 is used. (**B**) Peak number comparison of mzML, Numpress and Aird (6 decimal points stored), after ADAP chromatogram building in MZmine3. Vendor file 13 is used.

In order to verify the results in the different vendor files, we additionally compared the XIC results of file 7 and added a comparison with Numpress (see Fig. 5). Preprocessing workflow for LC–MS data can be accessed in the Supplementary. Result of SCIEX file 7 is the same as it of Thermo file 13. And comparison with Numpress, Aird (6 dp) exhibits better consistency with mzML (see Fig. 5B).

## Discussion

### Applicable file characteristics for SZDPD

Since we compress the tag array by manipulating each digit to form a byte array and then applying Zlib, the duplication of each byte is highest when the tag is 8-bit, that is, a byte storage, which is a favorable characteristic for the Zlib algorithm. Based on this, we see that 2^8^ spectra in a stack compress best in the test of number of spectra a stack contains. Given that, in our subsequent tests, we used the strategy of 256 spectra stacked.

We only test the files whose data acquisition modes are DDA and DIA, for the starting point of using SZDPD is based on the similarity of adjacent spectra. However, in other data acquisition modes like SRM, MRM and PRM, the similarity between adjacent MS1 and MS2 spectra is relatively small, thus there is no need to apply SZDPD. MS2 blocks in DDA files generally contains spectrums less than 256, therefore, we merely apply SZDPD to MS1 block for files in the DDA data acquisition mode.

Among the mainstream mass spectrometers that have been tested, files produce by SCIEX TOF6600 are the most suitable for application of SZDPD, followed by the SCIEX TOF5600 and Agilent 6550 iFunnel Q-TOF. Time of Flight (TOF) technology generate MS data with more similar adjacent spectra, and TOF6600 has the performance to deliver more sensitive, larger scale quantitative data, leading to the smaller and more repeatable deltas between m/z. As a result, stack compression offers more significant benefit.

### Portability of the SZDPD algorithm

In the SZDPD algorithm, the combined saved spectra are at the same MS level, and for MS2 spectra, they are required to come from the same MS1 window. In the Aird format, unlike mzML files, which arrange the spectra by rentention time, we reorder the mass spectra data, putting the spectra of MS1 together and the MS2 spectra from the same MS1 window together. Therefore, if other mass spectral file formats do not rearrange the spectrum, the use of the SZDPD algorithm will be limited.

### Superiority and development prospect of SZDPD

Cloud computing is growing in popularity, and MS data has been limited to large file sizes, leading to the difficulty to use the huge cloud server computing resources, especially data of proteomics. On the other hand, one of the important reasons why proteomics is difficult to build a Web-based analysis platform is that the huge data files lead to an unacceptable long analysis process, which is unfavorable to the Web-based analysis platform. Smaller files and faster data reading are the pursuits of many researchers who devote themselves to MS research. The application of ZDPD makes the mass spectral data greatly compressed, especially the data obtained by QE technique. By using SZDPD to compress the MS files, the compression level of mass spectrum data generated by TOF can reach about 30% while ensuring reasonable decompression time.

In the whole process of MS data from generation to analysis to results, information is gradually lost. What data needs to be stored and what data needs to be transmitted are interlinked. As a format intended to reduce data storage footprint and optimize the speed of data analysis, Aird (SZDPD) has favorable compression performance and appropriate data reading speed. Although file size has been remarkable compressed, there is still room of optimization in the data transmission of mass spectrum files in the calculation process. More effort is needed to explore suitable data for various uses.

## Methods

### Stack-ZDPD algorithm description

**Figure 6:**
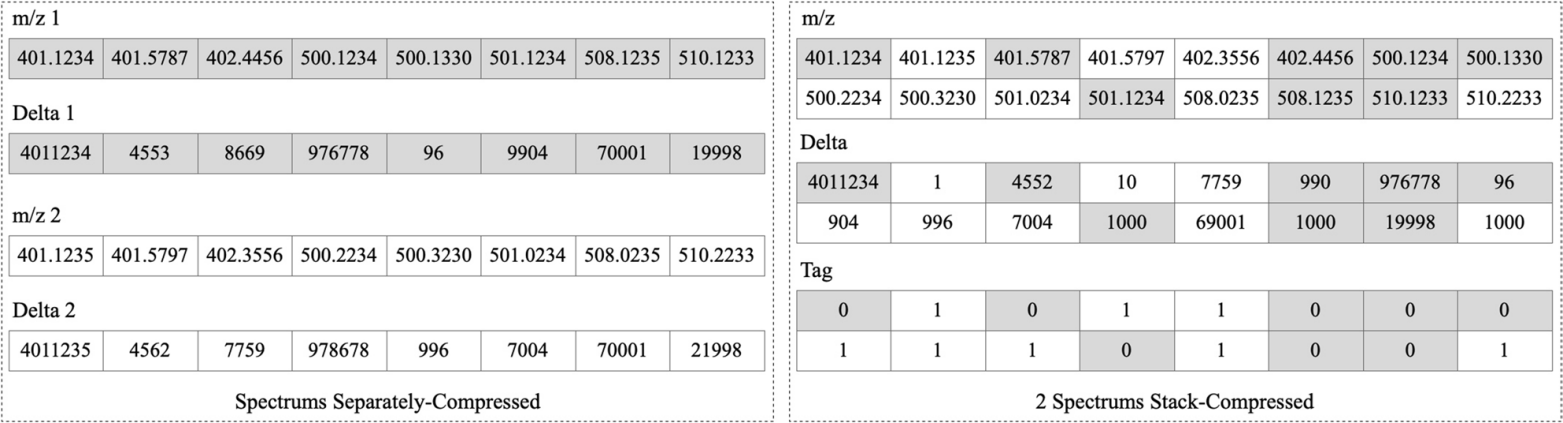
Schematic diagram of Stack-ZDPD compression principle. Floating-point m/z array is converted to ordered integer array, and then replace the later integer with delta of adjacent integers to use pFor library to compress the array. In principle of Stack-ZDPD, we merge several arrays at first and repeat the ZDPD process. Integer from spectrum 1 is in the gray cell, while integer from spectrum 2 is in the white cell. To restore the original arrays, corresponding spectrum tag should be stored. In the given example in the diagram, spectrum 1 is marked as “0”, and spectrum 2, “1”. The computer performs calculation in binary, thus tag of 2^n^ spectrums require n bits for storage.

A concise schematic diagram of the Stack-ZDPD algorithm is shown in Fig. 6. Some characteristics of m/z array can be obtained from experimental mass spectrum data: (a) In DDA mode, the range of fragment mass is about 0–6000, and for SWATH/DIA, the range is 400–12,000. Taken all, whether in metabolomics or proteomics, each m/z can be stored as integer type. (b) Numbers in m/z array are ordered for incrementation, one of the premises of using pFor library. (c) There are similar m/z in adjacent spectra, and deltas of merged array is relatively smaller and more repeated. Given that, the pFor compression algorithm performs better on merged arrays, providing room for compression optimization.

Data precision adjustment is achieved by enlarging m/z by different multiples. For example, 1 ppm (10 Daltons to calculate) corresponds to m/z-accuracy of 4 decimal places (dp). Therefore, a floating-point m/z of 1 ppm is multiplied by 10^4^ to store it as an integer. And for 0.1 ppm, the number is 10^5^.

### MS-data structure adjustment

**Figure 7:**
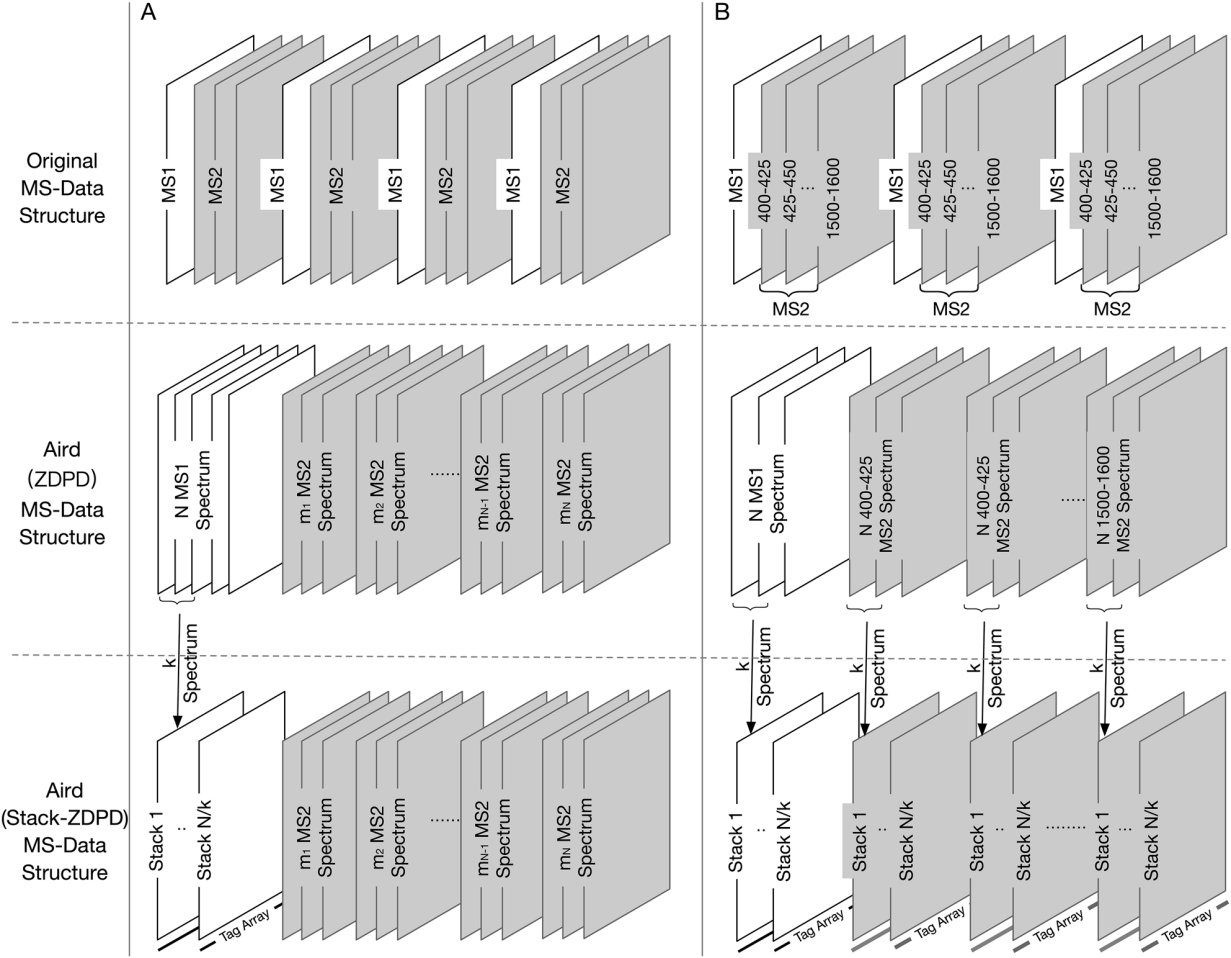
(**A**) MS-Data structures in data acquisition mode of DDA. In DDA mode, each MS1 spectra is originally followed by several relevant MS2 spectra. In data organization of Aird (ZDPD), MS1 spectra are gathered as MS1 block to accelerate the extracted ion chromatogram (XIC) calculation. According to Stack-ZDPD, each k spectra are combined before compression, so the output data is composed of several stacks, the merged spectrums. Each stack is stored along with a Zlib-compressed tag array for decoding. It should be noted that the number of spectra mi in the MS2 block is often not very large, thus MS2 data keeps the structure in Aird (ZDPD). (**B**) MS-Data structures in SWATH/DIA mode. Different from DDA mode, each MS2 spectra in DIA is set to a certain m/z SWATH window and MS2 data in the same window is generally extracted for analysis. Therefore, MS2 data is stored as MS2 blocks according to its m/z SWATH window and spectrum number of each MS2 block is theoretically same as MS1 block. Further, data in each block is stored as stacks to achieve better compression performance.

MS-Data structures of mzML, Aird (ZDPD) and Aird (Stack-ZDPD) are shown in Fig. 7. For each spectrum, its scanning number and start position are stored in order of RT in metadata of mzML, along with MS-data arranged according to the original RT. In addition to the classical index strategy, we rearrange the spectra, and store the MS1 and MS2 spectra into MS1 and MS2 blocks respectively, according to the calculation requirements instead of RT. Further, Stack-ZDPD is applied for each MS-data block. Storage of the chromatogram and spectral data (scan start times, m/z, and intensities) is flexible and self-described in terms of floating-point precision and layout.

### Material

To verify Aird (Stack-ZDPD) across a broad range of proteomics and metabolomics data sets generated in different laboratories, we select a wide variety of MS technologies and instruments from mainstream vendors. The data sets are depicted in Table 1. We test Aird (Stack-ZDPD) among different MS types, including SWATH-DIA, AIF-DIA and DDA, and from the major vendors including Thermo, Agilent, and SCIEX.

Our implementation of Stack-ZDPD has been integrated on the followed two tools, available from https://github.com/CSi-Studio/Aird-SDK and https://github.com/CSi-Studio/AirdPro. (a) Aird SDK: The SDK of Aird data accessing for both Java and C# languages. This enables third-party bioinformatics tool developers to import and export data written in Aird simply using library available in Java. (b) Aird Pro: A GUI client for Aird data converting. Hence, the proprietary raw vendor files can be directly converted into Aird (Stack-ZDPD) using the "Aird Pro" tool and MS data is conveniently accessed through Aird SDK.

The vendor file reader API is from MSConvert. Files are transformed into mzML, Numpress, mzMLb using the following command line code. (a) msconvert < Input file >. (b) msconvert < Input file > --mzML --zlib --mz64 --inten32 –n. (c) msconvert --mzMLb -z --mzLinear --mzTruncation = 19 --intenTruncation = 7.

## Supplementary Information

Supplementary Information.

## Data Availability

Aird SDK allows scholars to access MS data stored in Aird (SZDPD) efficiently, available at https://github.com/CSi-Studio/Aird-SDK. AirdPro can convert vendor files into Aird files, available at https://github.com/CSi-Studio/AirdPro.
