# Author Correction: StackZDPD: a novel encoding scheme for mass spectrometry data optimized for speed and compression ratio

**DOI:** 10.1038/s41598-022-12510-z

**Published:** 2022-05-19

**Authors:** Jinyin Wang, Miaoshan Lu, Ruimin Wang, Shaowei An, Cong Xie, Changbin Yu

**Affiliations:** 1 Zhejiang University, Hangzhou, 310058 China; 2 School of Life Science, Westlake University, Hangzhou, 310023 China; 3 School of Engineering, Westlake University, Hangzhou, 310023 China; 4 Fudan University, Shanghai, 200438 China; 5 College of Artifcial Intelligence and Big Data for Medical Science, Shandong First Medical University, Jinan, 250117 China; 6 Carbon Silicon (Hangzhou) Biotechnology Co., Ltd, Hangzhou, 310030 China

Correction to: *Scientific Reports* 10.1038/s41598-022-09432-1, published online 30 March 2022

In the original version of this Article, Jinyin Wang was incorrectly listed as a corresponding author. The correct corresponding author for this Article is Changbin Yu. Correspondence and request for materials should be addressed to Yucb@sdfmu.edu.cn

In addition, there was an error in the spelling of the author Ruimin Wang which was incorrectly given as Ruiming Wang.

The original version of this Article also contained an error in Figure 7 where “Spectrum” was incorrectly given as “Spectrums”. The original Figure 7 and accompanying legend appear below.

**Figure 7:**
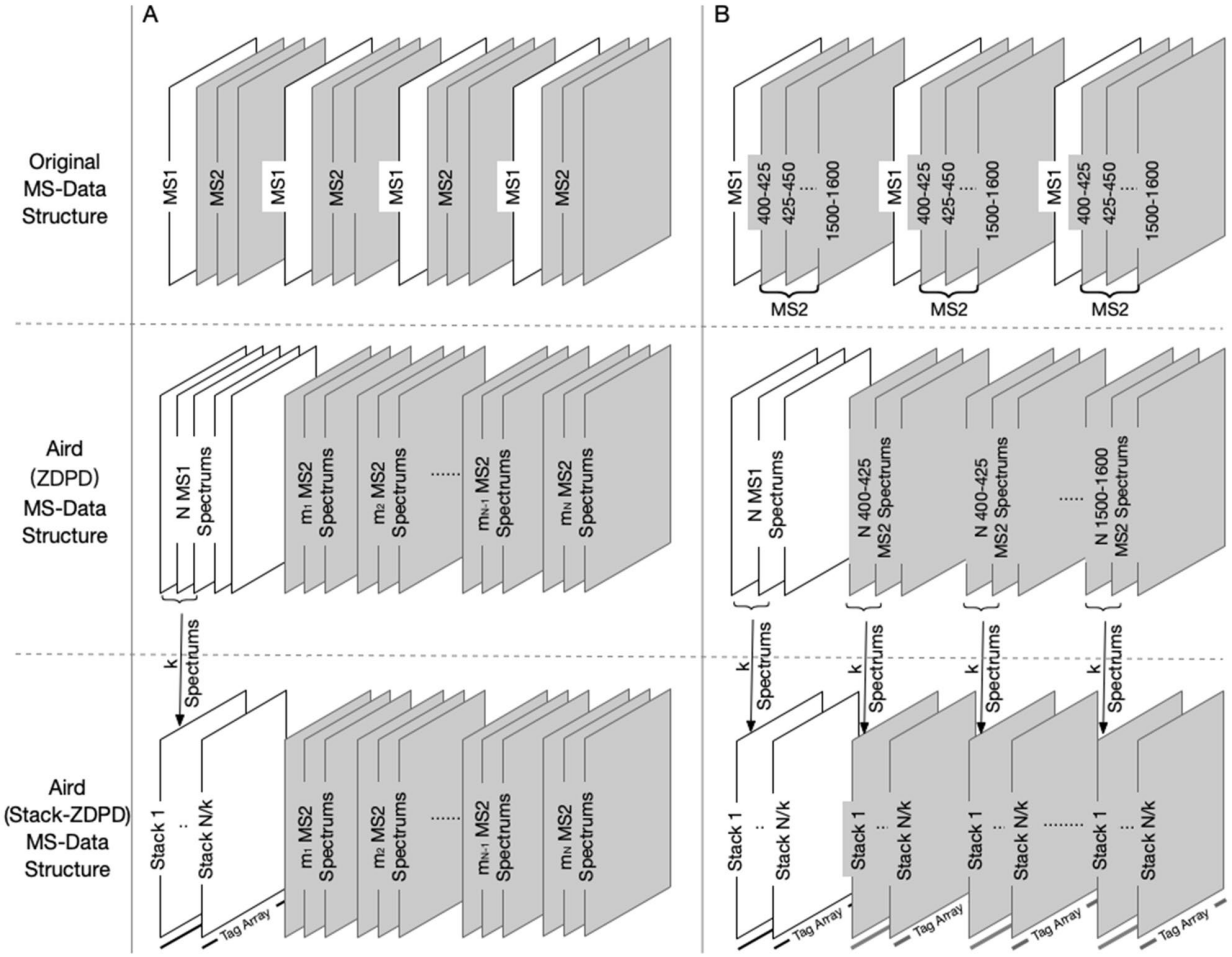
(**A**) MS-Data structures in data acquisition mode of DDA. In DDA mode, each MS1 spectra is originally followed by several relevant MS2 spectra. In data organization of Aird (ZDPD), MS1 spectra are gathered as MS1 block to accelerate the extracted ion chromatogram (XIC) calculation. According to Stack-ZDPD, each k spectra are combined before compression, so the output data is composed of several stacks, the merged spectrums. Each stack is stored along with a Zlib-compressed tag array for decoding. It should be noted that the number of spectra mi in the MS2 block is often not very large, thus MS2 data keeps the structure in Aird (ZDPD). (**B**) MS-Data structures in SWATH/DIA mode. Different from DDA mode, each MS2 spectra in DIA is set to a certain m/z SWATH window and MS2 data in the same window is generally extracted for analysis. Therefore, MS2 data is stored as MS2 blocks according to its m/z SWATH window and spectrum number of each MS2 block is theoretically same as MS1 block. Further, data in each block is stored as stacks to achieve better compression performance.

The original Article and accompanying Supplementary Information file have been corrected.

