# Supplementary material for: StackZDPD: a novel encoding scheme for mass spectrometry data optimized for speed and compression ratio
Source: Sci Rep. 2022 Mar 30;12:5384. doi: 10.1038/s41598-022-09432-1 (PMC8967824; doi:10.1038/s41598-022-09432-1)
Supplement: Supplementary file 1 — Supplementary Information. [file 41598_2022_9432_MOESM1_ESM.pdf]

StackZDPD, A Novel Encoding Scheme for Mass Spectrometry Data Optimized for Speed and Compression Ratio

Jinyin Wang[1,2,6], **Miaoshan Lu**[1,3,6], **Ruimin Wang**[3,4,6], **Shaowei An**[2,4,6], **Cong Xie**[5,6], **& Changbin Yu**[5,6,*]

1. Zhejiang University, Hangzhou, 310058, China
2. School of Life Science, Westlake University, Hangzhou, 310023, China
3. School of Engineering, Westlake University, Hangzhou, 310023, China
4. Fudan University, Shanghai, 200438, China
5. College of Artificial Intelligence and Big Data for Medical Science, Shandong First Medical University, Jinan, 250117, China
6. Carbon Silicon (Hangzhou) Biotechnology Co., Ltd, Hangzhou, 310030, China

* Yucb@sdfmu.edu.cn

Supplementary

Preprocessing workflow for LC-MS data

Import raw data

Raw data files are imported into MZmine 3 using the raw data methods, and drop-down menu is showed in Figure S1. We adapted the aird format of mass spectrometry file for MZmine 3 and "aird" method can be found in the "Raw data import" menu. For comparation with mzML format and data compressed with Numpress, the other two formats of raw data are imported using the "mzML" method.

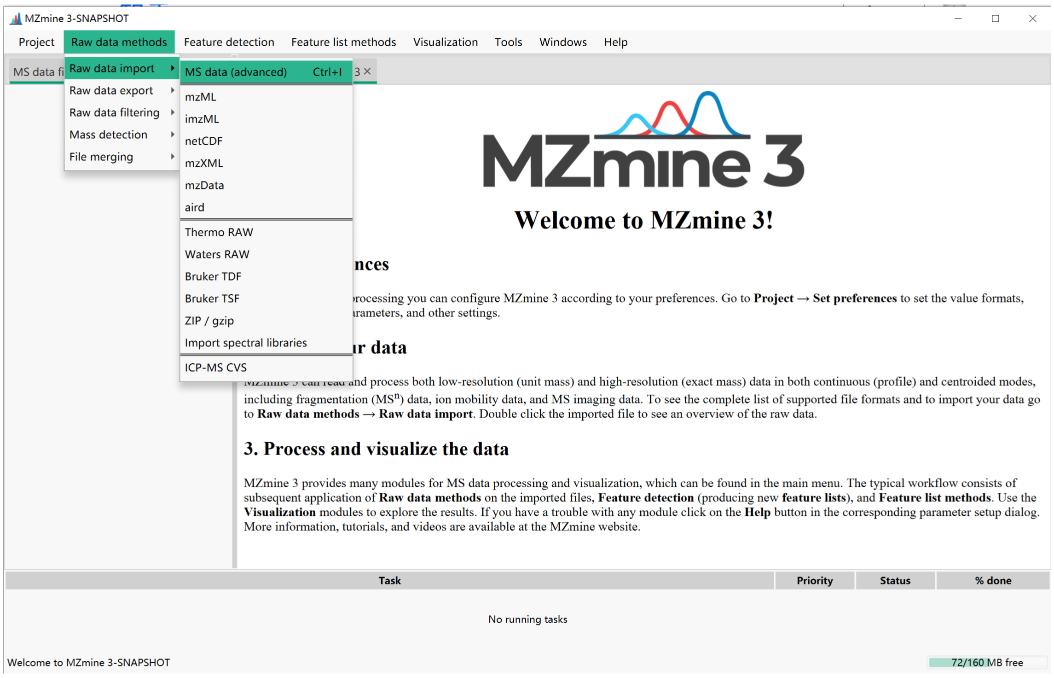

Figure S1. Import raw data into MZmine 3. Imported raw data files are listed under "MS data files" on the left sidebar.

Detect masses from profile mass spectra

The mass detection step detects mass centroids from profile mass spectra. The "Exact Mass" detector is suitable for high-resolution MS data.

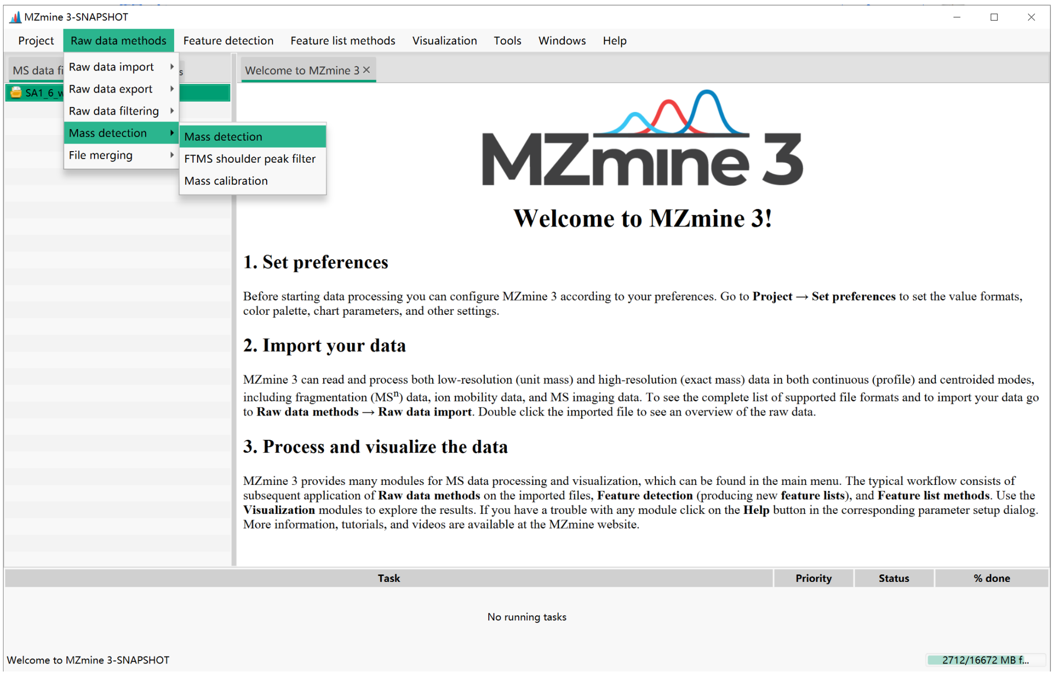

(A)

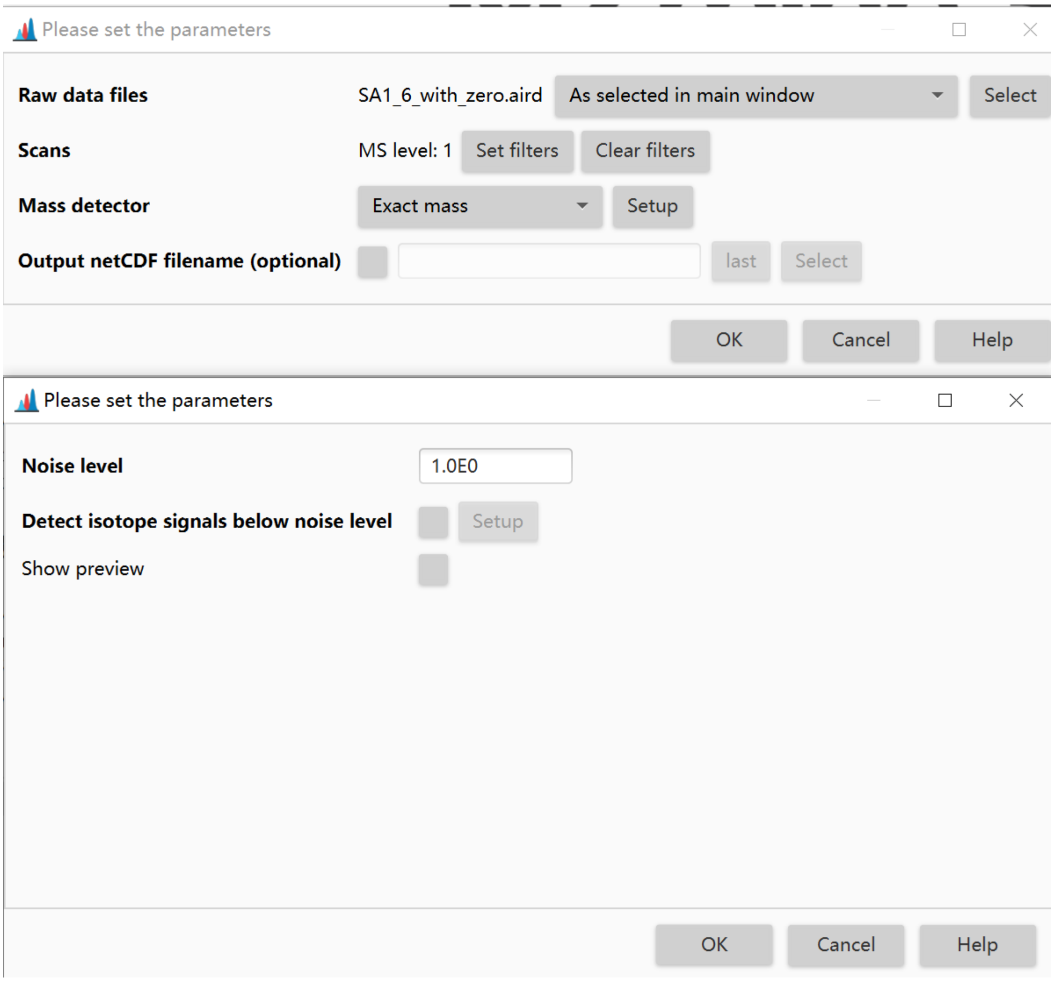
Please set the parameters
Raw data files
SA1_6_with_zero.aird
As selected in main window
Select
Scans
MS level: 1
Set filters
Clear filters
Mass detector
Exact mass
Setup
Output netCDF filename (optional)
last
Select
OK
Cancel
Help
Please set the parameters
Noise level
1.0E0
Detect isotope signals below noise level
Setup
Show preview
OK
Cancel
Help

(B)

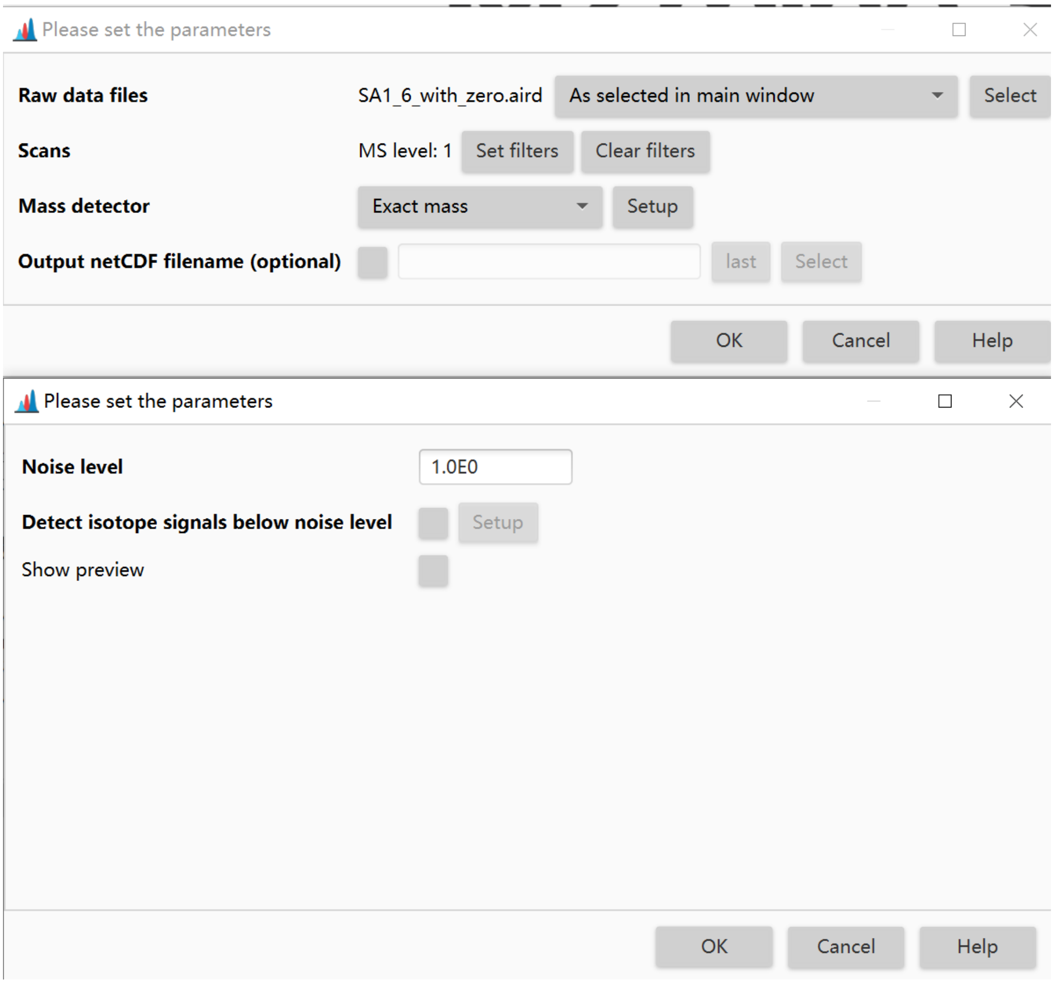
Please set the parameters
Raw data files
SA1_6_with_zero.aird
As selected in main window
Select
Scans
MS level: 1
Set filters
Clear filters
Mass detector
Exact mass
Setup
Output netCDF filename (optional)
last
Select
OK
Cancel
Help
Please set the parameters
Noise level
1.0E0
Detect isotope signals below noise level
Setup
Show preview
OK
Cancel
Help

(C)

Figure S2. Mass detection in MZmine 3. (A) The mass detection method can be accessed via the "Raw data methods" drop-down menu. (B) "Exact mass" is one of the mass detection methods. (C) Parameters used in "Exact mass".

Construct EIC by ADAP

ADAP is recommended by the manual of MZmine 3 for LC-MS data especially data generated by mass analyzers whose mass measurement accuracy tends to be higher for more intense signals.

ADAP chromatogram builder can be found in the drop-down menu of "Feature detection". As a result of the EIC construction, a list of EICs is produced (Figure S3C).

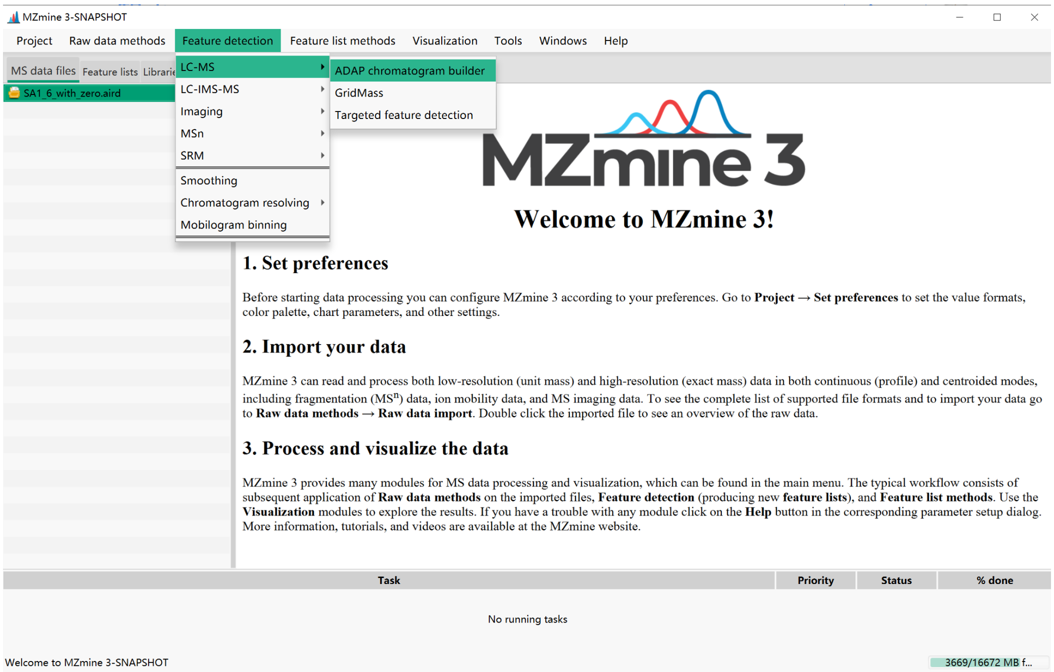

(A)

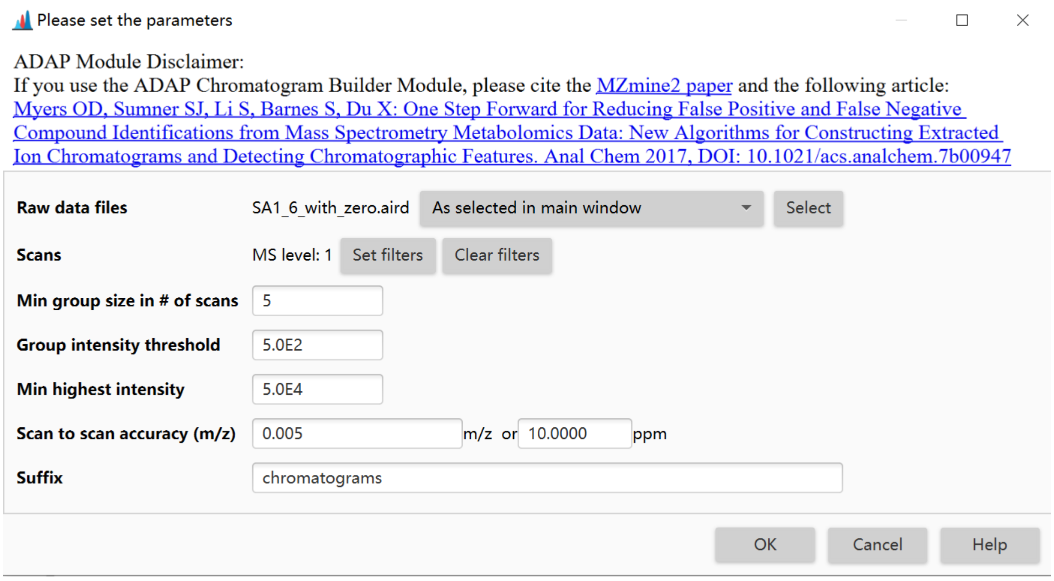

(B)

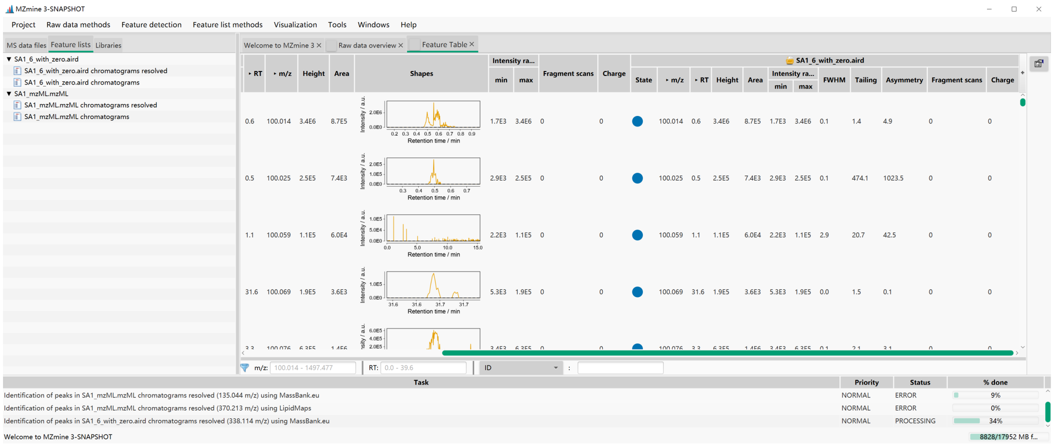

(C)

Figure S3. ADAP chromatogram builder in MZmine 3. (A) Methods location in MZmine 3. (B) Parameters applied in ADAP builder. (C) Results of EIC construction.

Peak detection

After EICs have been constructed, ADAP detects chromatographic peaks from each of these EICs. The used ADAP peak detection method is accessed via "ADAP resolver" in the "Chromatogram resolving" drop-down menu (Figure S4A). After peak detection, a new feature list with suffix of "resolved" is added to the "Feature lists" column.

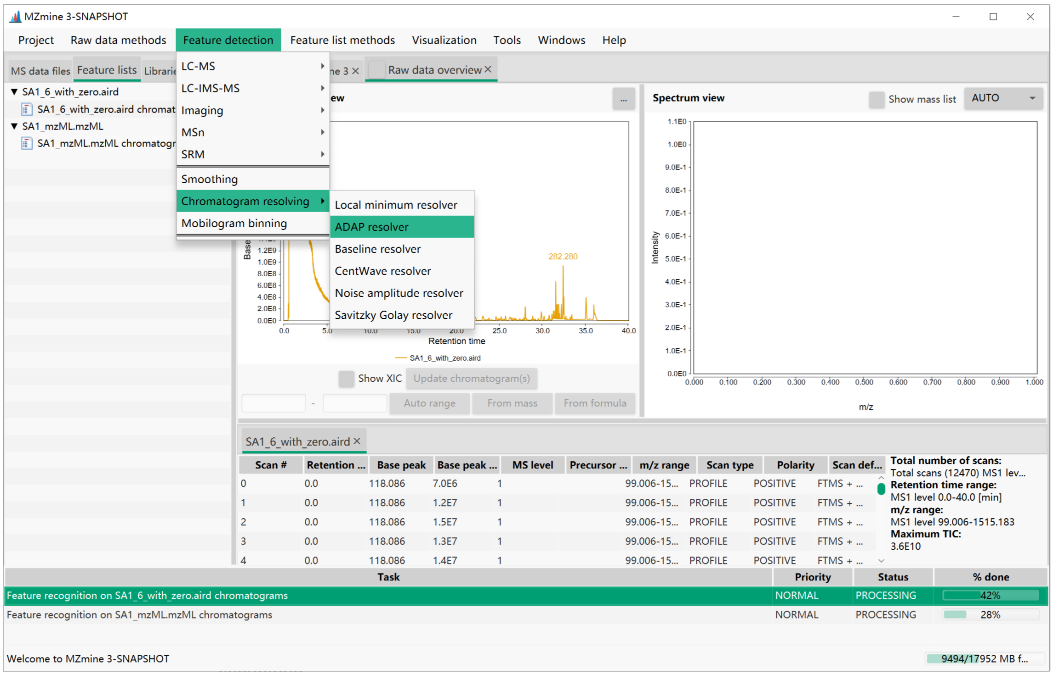

(A)

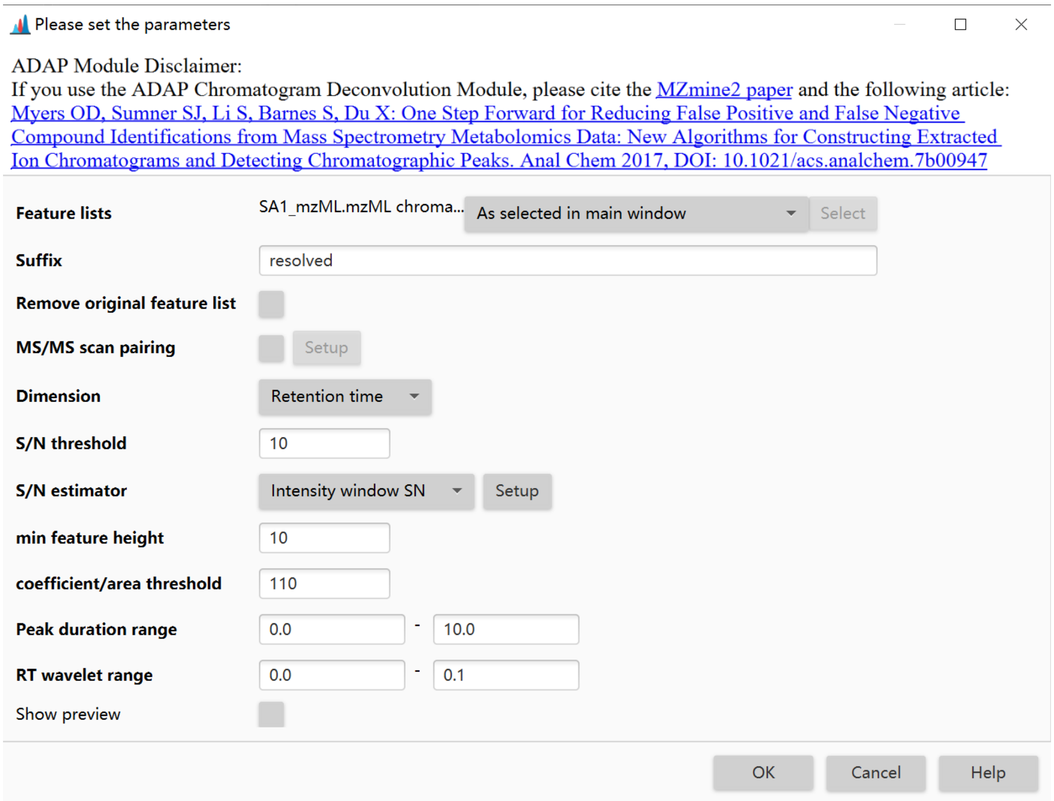

(B)

Figure S4. Detect chromatographic peaks by ADAP. (A) "ADAP resolver" method location. (B) Parameters used in peak detection.

Alignment

Alignment intends to identify corresponding peaks across samples. Alignment methods can be accessed through the "Feature list methods" drop-dwon menu in MZmine 3.

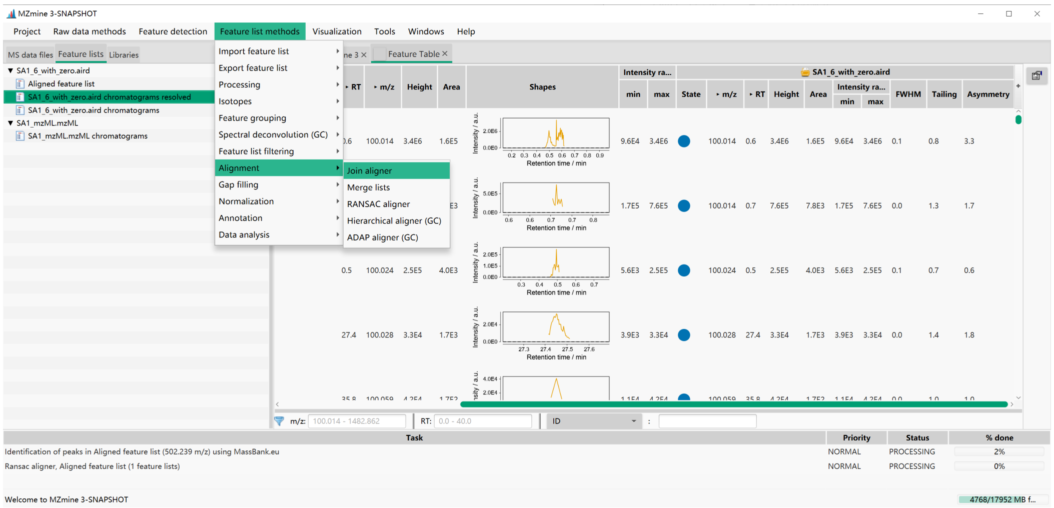

(A)

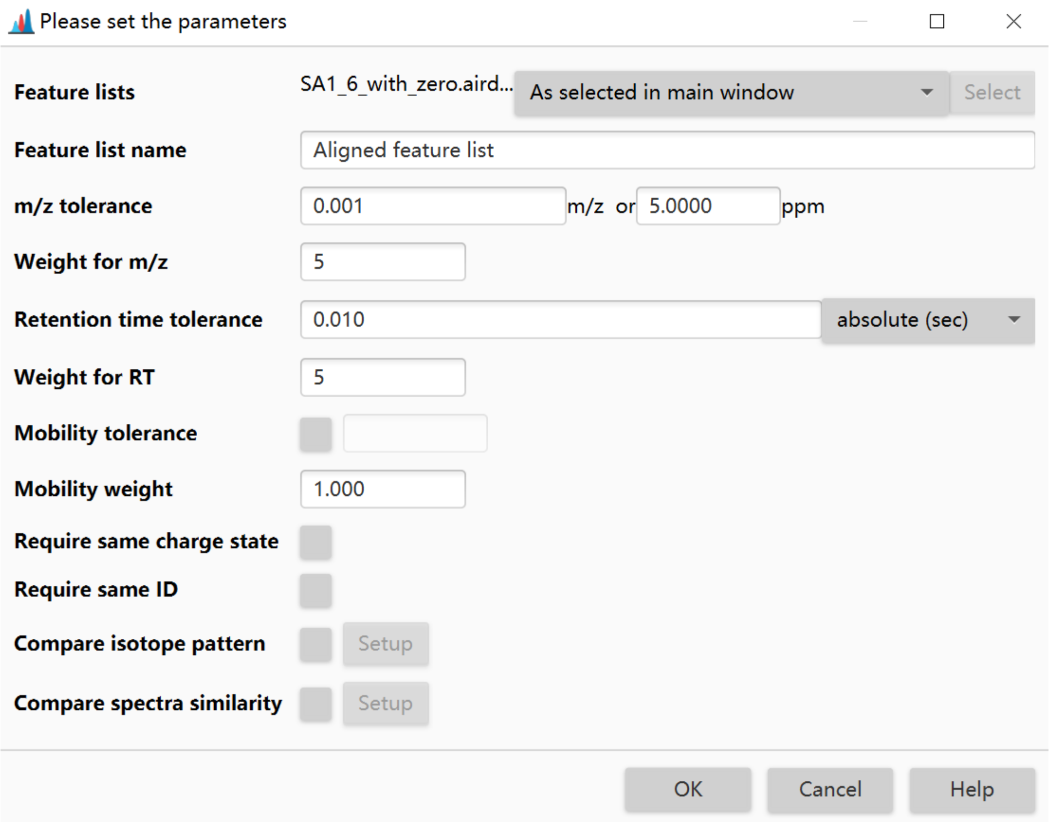

(B)

Figure S5. Alignment in MZmine 3. (A) Alignment methods can be accessed through the "Feature list methods" drop-down menu. (B) Parameters used in "Join aligner".
